# Guardians and Mediators of Metastasis: Exploring T Lymphocytes, Myeloid-Derived Suppressor Cells, and Tumor-Associated Macrophages in the Breast Cancer Microenvironment

**DOI:** 10.3390/ijms25116224

**Published:** 2024-06-05

**Authors:** Maria Rosaria Ruocco, Armando Gisonna, Vittoria Acampora, Anna D’Agostino, Barbara Carrese, Jessie Santoro, Alessandro Venuta, Rosarita Nasso, Nicola Rocco, Daniela Russo, Annachiara Cavaliere, Giovanna Giuseppina Altobelli, Stefania Masone, Angelica Avagliano, Alessandro Arcucci, Giuseppe Fiume

**Affiliations:** 1 Department of Molecular Medicine and Medical Biotechnology, University of Naples Federico II, 80131 Naples, Italy; mariarosaria.ruocco2@unina.it (M.R.R.); armando.gisonna@unina.it (A.G.); 2 Department of Public Health, University of Naples Federico II, 80131 Naples, Italy; vittoria.acampora@unina.it (V.A.); alessandro.venuta@unina.it (A.V.); angelica.avagliano@gmail.com (A.A.); 3 IRCCS SYNLAB SDN, Via Emanuele Gianturco 113, 80143 Naples, Italy; anna.dagostino@synlab.it (A.D.); barbara.carrese@synlab.it (B.C.); jessie.santoro@synlab.it (J.S.); 4 Department of Movement Sciences and Wellness, University of Naples “Parthenope”, 80133 Naples, Italy; rosaritanasso@gmail.com; 5 Department of Advanced Biomedical Science, University of Naples Federico II, 80131 Naples, Italy; nicola.rocco@unina.it (N.R.); daniela.russo@unina.it (D.R.); giovannagiuseppina.altobelli@unina.it (G.G.A.); 6 Plastic Surgery Unit, University of Naples Federico II, 80131 Naples, Italy; annachiaracavaliere@yahoo.it; 7 Department of Clinical Medicine and Surgery, University of Naples Federico II, 80131 Naples, Italy; stefania.masone@unina.it; 8 Department of Experimental and Clinical Medicine, University of Catanzaro “Magna Graecia”, 88100 Catanzaro, Italy; fiume@unicz.it

**Keywords:** breast cancers, tumor microenvironment, tumor-infiltrating lymphocytes, tumor-associated macrophages, myeloid-derived suppressor cells, metastasis

## Abstract

Breast cancers (BCs) are solid tumors composed of heterogeneous tissues consisting of cancer cells and an ever-changing tumor microenvironment (TME). The TME includes, among other non-cancer cell types, immune cells influencing the immune context of cancer tissues. In particular, the cross talk of immune cells and their interactions with cancer cells dramatically influence BC dissemination, immunoediting, and the outcomes of cancer therapies. Tumor-infiltrating lymphocytes (TILs), tumor-associated macrophages (TAMs), and myeloid-derived suppressor cells (MDSCs) represent prominent immune cell populations of breast TMEs, and they have important roles in cancer immunoescape and dissemination. Therefore, in this article we review the features of TILs, TAMs, and MDSCs in BCs. Moreover, we highlight the mechanisms by which these immune cells remodel the immune TME and lead to breast cancer metastasis.

## 1. Introduction

Breast cancers (BCs) are the most commonly diagnosed malignancies worldwide and are very heterogeneous solid tumors classified by immune-histochemical studies into three major types: estrogen receptor (ER) and progesterone receptor (PR) positive, human epidermal growth factor receptor 2 positive (HER-2^+^), and triple-negative breast cancers (TNBCs) [1]. BCs expressing both ER and PR represent approximately 85% of all BCs [2]. Furthermore, ER and PR BCs are further divided into two subtypes: luminal A and luminal B. In particular, luminal A are BCs that are ER^+^ and/or PR^+^ and HER2^−^ that are characterized by low expression of the Ki-67 proliferation marker. Luminal B includes BCs that are ER^+^ and/or PR^+^, HER2^+^ or HER2^−^, and that show high Ki-67 expression and worse prognosis than luminal A. Both HER2^+^ BCs and TNBCs comprise about 15% of BCs [2]. HER2^+^ BCs overexpress the HER-2 receptor, which modulates cell growth and differentiation, exhibit high Ki-67 expression, and are frequently associated with mutations of the *p53* gene. This BC type is an aggressive tumor that is correlated with diminished survival, enhanced risk of disease recurrence, and enhanced likelihood of metastasis [2]. TNBCs do not express any receptor, are they the most heterogeneous type of BCs, have a high risk of recurrence, have a higher capability to metastasize, and give shorter overall survival compared to the other types of BCs [1,3]. All BCs consist of heterogeneous tissues characterized by cancer cells and a tumor microenvironment (TME) that dramatically regulates the metastatic process, causing more than 90% of BC-related deaths and influencing therapeutic outcomes [4].

In particular, the TME includes the extracellular matrix (ECM), blood and lymphatic tumor vessels, non-cancerous stromal cells, immune cells such as tumor-infiltrating lymphocytes (TILs), tumor-associated macrophages (TAMs), and myeloid-derived suppressor cells (MDSCs) [3,4]. Among TME immune cells, CD8^+^ cytotoxic T cells, CD4^+^ T cells, TAMs, and MDSCs can be considered the major players in the immunological milieu of the BC microenvironment that significantly influence BC outcomes and clinical therapeutic results [4,5]. This review focuses on the pivotal roles played by T lymphocytes, TAMs, and MDSCs in maintaining homeostasis within the BC immune microenvironment and influencing the metastatic cascade. Understanding the dynamic interplay of T lymphocytes, TAMs, and MDSCs in the BC immune microenvironment holds promise for developing targeted therapeutic strategies to tip the balance in favor of anti-tumor immunity and restrain the metastatic process.

### 1.1. Tumor-Infiltrating Lymphocytes

#### 1.1.1. T lymphocyte Development and Differentiation

T lymphocytes, commonly referred as T cells, constitute a fundamental component of the adaptive immune system, playing a pivotal role in orchestrating cell-mediated immune responses that are essential for maintaining overall host health and defending against a multitude of diseases. T cells are categorized into various subsets, including CD4^+^ and CD8^+^ αβ T cells, as well as additional populations like γδ T cells and natural killer T (NKT) cells [6]. These T cells originate from thymocyte progenitors derived from the bone marrow and the thymus. Within the thymus, T cells undergo a series of developmental stages, transitioning from double-negative (CD4^−^CD8^−^, DN) to double-positive (CD4^+^CD8^+^, DP) and finally to single-positive (CD4^−^CD8^+^ or CD4^+^CD8^−^ SP) stages [7,8,9]. During the DN stage, thymocytes progress through four distinct stages (DN1 to DN4) based on CD44 and CD25 expression within the lineage-negative population [10,11]. At the DN3 stage, the genes coding for T cell recombination-activating 1 and 2 proteins (RAG1 and RAG2) become activated in the thymic cortex, initiating the random recombination of V, D, and J segments. This recombination process leads to the expression of a pre-TCR (comprising α and β chains) [12,13]. Thymocytes expressing a functional pre-TCR, constituted by TCRβ together with pTα (invariant pre-TCRα) and CD3 molecules, undergo β-selection and differentiate into αβ T cells [14]. Alternatively, thymocytes may differentiate into γδ T cells. Thymocytes failing to generate a productive TCR rearrangement undergo apoptosis [14]. Thymocytes expressing a fully functional αβTCR interact with MHC complexes of cortical thymic epithelial cells (cTECs) [15]. Those with moderate affinity to peptide-MHC undergo positive selection and progress to the single-positive (SP) stage [16]. Thymocytes with strong affinities to self-peptides presented by cTECs trigger apoptosis (negative selection) or differentiate into regulatory T (Treg) cells in the thymic medulla [17]. Following positive and negative selection, double-positive (DP) cells differentiate into either CD4^+^ SP cells, as regulated by a strong TCR signal and Thpok, or CD8^+^ SP cells, which is regulated by a weak TCR signal and Runx3 [6,9]. Single-positive T cells then exit the thymus and enter the bloodstream.

#### 1.1.2. CD8^+^ T Cells

**Figure 1:**
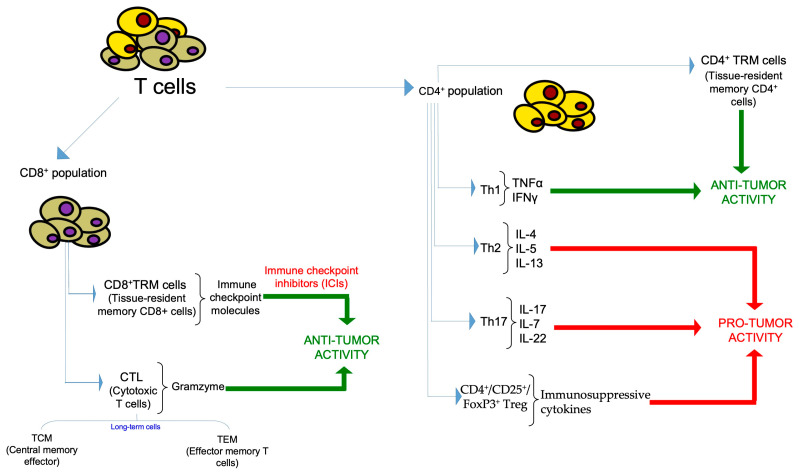
A schematic representation of tumor-infiltrating lymphocytes (TILs). TILs consist of two main cell populations, represented by CD4^+^ cells and CD8^+^ cells, which recognize cognate antigenic peptides presented by MHC class II and I molecules, respectively. In turn, CD4^+^ cells and CD8^+^ cells comprise other functionally different subpopulations, displaying pro-tumor or anti-tumor activities. CD4^+^CD25^+^FoxP3^+^ human regulatory T (Treg) cells represent one of the main immunosuppressive subsets of CD4^+^ T cells. The figure shows molecules produced by these T cell subpopulations that are associated with pro-tumor or anti-tumor functions.

Immune cells help to eliminate the emerging tumor cells but they can also facilitate tumor development and metastasis and contribute to therapy resistance [18,19]. Furthermore, the interaction between cancer cells and the TME strongly affects anti-tumor immunity [20]. However, the interplay between immune and tumor cells is regulated by intricate mechanisms that also involve cell metabolic reprogramming that can influence the suppression and/or the activation of anti-tumor immunity [21]. BC is not considered a tumor predisposed to treatment with immunotherapy because of its low mutational load as compared with other cancer types [22]. Recently, many studies have described a complex immune environment in some BC subtypes, which is characterized mainly by tumor-infiltrating lymphocytes (TILs) and tumor-infiltrating immune cells (TIICs) as well as tertiary lymphoid structures (TLS) [23,24,25]. In particular, TILs include T cells, B cells, and natural killer (NK) cells, which in BC represent about 75%, 20%, and 5%, respectively, of TILs [26]. The single subtypes of immune infiltrates play a different role in the TME, acting as negative or positive modulators of tumor growth, progression, metastasis, and therapy responses. Hence, the location and characterization of infiltrating immune subtypes in the BC microenvironment are critical for evaluating tumor prognosis and therapy responses [27]. Nonetheless, the characterization of TILs (Figure 1) and their prognostic utility in BCs has been conflicted because of the variability in the methods and criteria used to quantify TILs [28].

A high density of CD8^+^ cytotoxic T lymphocytes (CTL) in TIL populations usually is associated with a better prognosis in BCs, independent of other prognostic markers [29]. In particular, CTL recognize and bind to tumor cells and release granzyme that activates an apoptotic program in cancer cells [27]. However, these early-effector CTLs can differentiate and survive long term as central memory T cells (TCM) and effector memory T cells (TEM) [30].

It is known that the efficacy of immune checkpoint inhibitor (ICI) therapy is associated with high intra-tumoral, stromal, or invasive marginal levels of CD8^+^ TILs [31]. A study conducted in a total of 3837 BC patients, using an xCell-gene-signature-based method on whole tumor gene expression data, demonstrated a high CD8 score in TNBCs that was associated with high tumor immune cytotoxic activity, high immunoreactivity, and better survival. The authors of this study suggest that a high CD8 score in TNBCs can also be used as a predictive marker for treatment response to ICIs [28].

Recently, efficacy has been shown for ICIs in association with standard chemotherapy in TNBCs [32,33]. The pre-existence of PD-L1-positive cells is pivotal for the response to ICIs in patients with advanced TNBCs [34]. A population of memory T cells, named tissue-resident memory T (TRM) cells, resides in the tissues and does not recirculate, forming the first line of defense against pathogens [35]. These cells express high levels of immune checkpoint molecules, and their presence in various human cancers, including BCs, is linked to a better tumor prognosis [36]. It has been shown that the use of anti-PD-1 antibodies on TRM cells isolated from human lung cancer promotes cytolytic activity toward autologous tumor cells [37]. The presence of CD8^+^TRM cells is very important for BC immune surveillance, especially during post-immune checkpoint blockade. Indeed, it has been shown that CD8^+^TRM cells localized in TNBC tissues after tumor clearance can locally expand and exert a pivotal protective role against tumor reactivation, with minimal contribution from circulating T cells [38].

Tumors, including BCs, implement mechanisms to evade or reduce the CD8^+^ anti-cancer activity. It has been demonstrated that the level, phenotype, and distribution of immune cells within the TME are key determinants during immune checkpoint blockade (ICB) responses [39,40].

Cancer-associated fibroblasts (CAFs) represent major constituents of the BC TME, and they participate in tumor growth, progression and therapy resistance [41,42]. This heterogeneous stromal cell population plays a key role in modulating anti-tumor immunity and contributes to the generation of an immunologically cold tumor phenotype. In particular, the cold tumor phenotype is characterized by an immune microenviroment that does not lead to a strong immune response [43]. The CAF secretome exerts an immunomodulatory action by regulating immune cell recruitment and functions within tumors [44,45,46] as well as CAFs contributing to construct an ECM that acts as physical barrier precluding the infiltration of immune cells into tumors [47,48,49]. CAFs targeting strategies can intensify anti-tumor immune response. Recently, it has been shown that a murine BC model with a high density of CAFs was characterized by an insensitivity to the ICB, due to a reduced level of CD8^+^ T cells. In particular, the authors demonstrated that in vivo depletion of a subset of CAFs expressing alpha smooth muscle actin (α-SMA) was associated with an increase in CD8^+^ T cell infiltration and enhanced sensitivity to ICB therapy [50]. Furthermore, a number of cytokines secreted by CAFs can modulate peripheral CD8^+^ T cell infiltration within tumors. In particular, it has been shown that CXCL12 regulates CD8^+^ T migration and localization in the stromal compartment surrounding the tumor, thereby decreasing CD8^+^ T cell infiltration within tumors [51,52].

However, several aspects, such as the heterogeneity of CAFs, their variability in different tumors, and the absence of specific CAF markers as well as a reduced number of in vivo experimental models, make it more difficult to interpret studies on the effects of CAFs during anti-tumor immunity [53]. Hence, analysis of different CAF subpopulations and their tumor localization can represent a valid tool for improving knowledge regarding the role of CAFs in the immunomodulation of the TME and to find new therapeutic targets.

It is crucial to acknowledge that the prognostic impact of CD8^+^ T cell infiltration within breast tumors is also contingent on their spatial distribution. In numerous cancers, the presence of CD8^+^ TRM cells within the TME is linked to a favorable survival prognosis [54]. In particular, the spatial localization of CD8^+^TRM cells in breast tumor tissues represents a key factor in clinical outcome. With respect to other CD8^+^ TILs, the main characteristic of CD8^+^ TRM cells is represented by their capacity to localize near or among cancer cells. It has been demonstrated that a high density of CD8^+^TRM cells in cancer islands within breast tumors, rather than in overall tumor tissue or tumor stroma, is linked to relapse-free survival (RSF) [55]. Hence, the evaluation of CD8^+^TRM cells present within cancer islands can be a good predictive marker of the efficacy of different immune therapies.

Cyclooxygenase-2 (COX-2) and nitric oxide synthase-2 (NOS-2), which contribute to immune suppression within TME, represent other markers whose tumor expression can be predictive of the level of immune response and/or outcome [56,57]. COX-2 and NOS-2 expression in BCs modulate immune signaling and are associated with disease progression [56,57]. However, it is unclear how COX-2 and NOS-2 affect the spatial distribution of immune cells in tumors. In a recent work, Somasundarama et al. demonstrated that NOS-2/COX-2 levels influenced both the polarization and spatial location of lymphoid cells in a TNBC murine model. In particular, tumors with low expression of both NOS-2 and COX-2 showed a high infiltration of CD8^+^ T cells into the tumor core relative to tumors with elevated expression of these two markers. Furthermore, the author, by using a TNBC NOS-2^−^ murine model, demonstrated that treatment with indomethacin, a NSAID, increased the intratumor level of both CD4^+^ and CD8^+^ T cells relative to untreated controls, and, in particular, a marked concentration of CD8^+^ T cells in the tumor core [58]. It is known that the activation of the cGAS-STING pathway has a pivotal role in anti-tumor immune responses. However, the use of STING agonists in clinical trials have shown only modest effects [59,60]. It has been shown that the use of celecoxib, a selective COX-2 inhibitor, in combination with the STING agonist cyclic GMP-AMP (cGAMP) induced an anti-cancer immunity in mouse tumor models. In particular, celecoxib affects glucose metabolism in the TME by decreasing lactate efflux and thereby increasing CD8^+^ T cell activation [61].

It is noteworthy that TME can function as a metabolic barrier to CD8^+^ T cells and as a result can affect anti-tumor immunity [62]. Modifications in the glycolytic pathways in the TME alter amino acid and lipid metabolism, impairing nutrient supply for immune cells and causing cancer immune escape [63]. Amino acid depletion in TME, due to high energy requirement of tumor cells, hampers the growth, differentiation, and functions of TILs [64]. It has been demonstrated that preferential use of glutamine by TNBC cells leads to a reduced availability of this amino acid for TILs. On the other hand, the increase in glutamine availability in TME promoted CD8^+^ T cell activation in a mouse TNBC model [65]. Arginine is an essential amino acid that is involved mainly in the activation of immune T cells. Arginase I (ARGI) catalyzes the hydrolysis of L-arginine into L-ornitine and urea. This enzyme, which is overexpressed in several types of BCs, is responsible for reducing L-arginine levels in TME, causing the inhibition of anti-tumor immunity [66,67]. Tryptophan is another essential amino acid whose metabolism affects anti-cancer immunity. In particular, the catabolism of tryptophan exerts an inhibitory effect on T cell proliferation and activation [68]. In the BC TME, tryptophan cleavage reduces the levels of this amino acid and produces its catabolites, which collectively contribute to the inhibition of T cell activation [68]. Indoleamine 2,3-dioxygenase (IDO) is an enzyme that catalyzes the degradation of tryptophan to kynurenine. The anti-tumor cytotoxic effects of T cells were increased in a TNBC model after treatment with IDO enzyme inhibitors [69]. Thus, it can be assumed that the metabolism modulation of some amino acids can contribute to improvements in BC immunogenicity.

Lipid metabolism, particularly for fatty acids, is central in influencing immune activity or tolerance of immune cells [70]. The reprogramming of fatty acid metabolism represents a critical factor for the survival of BC cells [71] as well as for tumor immune surveillance [72]. Indeed, it has been demonstrated that obesity can be a predisposing factor for the development and progression of BC and, in particular, it contributes to tumor immune escape by reducing both the CD8^+^ T cell/Treg and M1/M2 macrophage ratios [73]. A prolonged obesity status is responsible for a leptin-mediated shift of CD8^+^ T cell metabolism from glycolysis to fatty acid oxidation. This process provokes a decrease in CD8^+^ T cell effector functions resulting in BC progression [72]. Furthermore, obesity, through leptin signaling, contributes to the growth and metastasis of BC by supporting immunosenescence but, paradoxically, T cell senescence resulting in an increase in PD-1 expression and dysfunction can better predispose obese subjects to checkpoint blockade therapy [74]. Hence, the reprogramming of immune TME metabolism exerts a profound effect on anti-cancer immunity and on T cell activities. Therefore, therapies affecting metabolic pathways can contribute to reverse immunosuppression and increase the success of immunotherapies aimed at hindering BC progression and metastasis.

#### 1.1.3. CD4^+^ T Cells

CD4^+^ T cells represent a heterogeneous cell population constituted mainly by Th1, Th2, Th17 and FoxP3^+^ Treg cells. CD4^+^ T cells represent a highly adaptable and multifunctional component of adaptive T cell immunity, working in tandem with their counterpart, the CD8^+^ cytotoxic T cells. Within the complex network of immune responses, CD4^+^ T cells undergo differentiation into various functional subtypes in response to context-dependent signals. In particular, Th1 cells are polarized by IL-12 and IFNγ and characterized by the production of IFNγ and TNF-α; Th2 cells are polarized by IL-4 and secrete IL-4, IL-5, and IL-13; Th17 cells are polarized by IL-6 and TGF-β, and produce IL-17; Tregs are induced by TGF-β and IL-2 and produce IL-10 and TGF-β [75]. This inherent flexibility enables CD4^+^ T cells to assume the pivotal role of central coordinators in orchestrating immune responses. In particular, they play a crucial role in promoting anti-tumor immunity through various mechanisms.

Their primary functions are to provide essential support for CTLs and to facilitate antibody responses. Additionally, CD4^+^ T cells contribute to the anti-tumor arsenal by secreting key effector cytokines, such as IFNγ and TNFα. In specific contexts, these cells can also exert direct cytotoxic effects against tumor cells. This intricate interplay underscores the significance of CD4^+^ T cells as central orchestrators of immune responses and highlights their diverse strategies to counteract tumorigenesis.

In the context of BCs, the heterogeneity of CD4^+^ T cell populations is associated with less important prognostic value compared to that of CTLs [76].

High expression of genes regulating Th17 immune responses is associated with an adverse prognosis, whereas high Th1 gene expression is linked to improved disease-free survival (DFS) [77,78]. It is noteworthy that Th17 immunity can also be associated with anti-tumor responses [79].

In tissues from BC patients, Th1 cells are reduced whereas Th2 cells are increased compared to healthy donors [80]. Moreover, the Th2 inflammatory response leads to tumor immunoescape and can contribute to BC development [81].

Interleukin-22 (IL-22) produced by Th17 cells, and in humans also by Th1 cells, sustains the epithelial-to-mesenchymal transition and cancer cell migration [82].

In a breast and lung cancer murine experimental model, Th cells produce IL-22 that sustains cancer cells’ CD155 expression, inducing internalization of NK cells activating receptor CD226. This process abolishes NK cell function and induces an immunosuppressive niche leading to lung metastases [83]. It is noteworthy that Th 17 cells have an important role in BC invasiveness and metastasis and that the pro-inflammatory cytokine IL-17, produced by Th 17 cells, is upregulated in metastatic BCs [84].

Recently, by using a T cell receptor α (TCR α) repertoire-deficient mouse model, Zhang et al. observed that CD4^+^ TRM cells could initiate a potent anti-tumor immune response associated with a significant inhibition of both melanoma and BC progression. This CD4^+^ TRM cell-initiated anti-tumor immunity was dependent on NK cells and IFN-γ, and the CD4^+^ TRM/NK cell axis could orchestrate the formation of TME, inhibiting the expansion of MDSCs [85].

#### 1.1.4. Regulatory T Cells

CD4^+^CD25^+^FoxP3^+^ human regulatory T (Treg) cells are an immunosuppressive subset of CD4^+^ T cells. Treg cells are characterized by the expression of the master transcription factor forkhead box protein P3 (FOXP3) [86,87]. The *FOXP3* gene represents a master regulator in the development, maintenance, and function of Treg cells; indeed, *FOXP3* gene mutations induce Treg cell deficiency [88]. CD4^+^ Treg cells originate from the bone marrow, and they develop in the thymus (tTreg) and also in peripheral tissues (pTreg) [89,90] after antigenic stimulation of CD4^+^ T cells and in the presence of cytokines such as TGF-β [91].

Treg cells are involved in the suppression of immune response, maintaining self-tolerance and thus preventing autoimmunity and allergy responses [92]. Treg cells, in the TME under inflammatory conditions, acquire a strong immune-suppressive function [93]. It is known that the presence of a high infiltrate of Treg cells contribute to the formation of a strong immunosuppressive TME [94]. It has been shown that tumor-infiltrating Treg cells represent a major obstacle to the success of immunotherapy against cancer because they support tumor development, promote tumor persistence, promote metastasis generation, and prevent effective anti-tumor immunity [95,96]. Treg cells can negatively act on most immune cells and their immunomodulatory activity is carried out through several mechanisms. Treg cells can exert cytotoxic effect on CD8^+^ T cells through granzyme and perforin upon direct cell to cell contact [97]. Treg cells produce immunosuppressive cytokines [98] and constitutively express the cytotoxic T lymphocyte associated protein 4 (CTLA-4) that modulates dendritic cells (DCs) maturation and suppresses T cell activation. CTLA-4 is able to bind to CD80 and CD86 on mature APCs with higher affinity with respect to CD28 expressed by CD8^+^ T cells, acting as competitive inhibitors of CD28 [99]. Hence, Treg cells represent a good target to improve anti-tumor immunity. High infiltration levels of Treg cells in biopsy samples of BCs is associated with disease progression and reduced relapse-free and overall survival [100]. The differentiation of naive CD4^+^ T cells into peripheral Treg cells is also modulated by tumor-associated macrophages (TAMs) through the expression of some cytokines, including CXCL1 [101,102]. The level of CXCL1 in BC tissue is higher with respect to that in normal breast tissue [103]. In a BC murine model, the oral administration of Aiduqing (ADQ) suppressed tumor growth and metastasis by affecting the immunosuppressive TME. In particular, ADQ, by exerting an inhibitory effect on the expression and secretion of CXCL1 from TAMs, provoked a reduction in the differentiation of naive CD4^+^ T cells into Tregs and increased the cytotoxic effects of CD8^+^ T cells [102]. Treg cells have a pivotal role in the development of BC metastasis. In particular, in BC mouse models Treg cells are involved in lung metastasis [104]. The roles of Treg cells in tumor promotion and metastasis are strictly linked to the expression of some chemokine receptors. In fact, Treg cell recruitment in the TME can be regulated through the interaction between chemokines and their receptors. High levels of CCR4-positive Tregs are detected in BC lung metastasis. In particular, CCR4 receptor-specific chemokines (CCL17 and CCL22) are secreted by BC metastatic lung tissues, resulting in CCR4^+^ Treg accumulation in lung tissue [105]. CCR5, a chemokine receptor highly expressed on Treg cells, positively regulates the onset and metastatic progression of BCs after binding with its ligand CCL5 [106]. CCR5^+^ Treg cells in the BC TME promote tumor metastasis to lymph nodes and bone marrow [107].

Moreover, in primary tumors the infiltration of Treg cells has been linked to the presence of circulating tumor cells, thus indicating that Treg cells could have a role in the tumor cells’ dissemination [108]. It has been shown that the evaluation of Treg cell infiltration in distant BC metastasis can represent a tool to identify patients with a lower likelihood of 2-year survival [109]. In particular, FoxP3 has a pivotal role in BC and could represent a good predictive factor in BC progression and invasiveness [38,110]. In a study conducted in a group of early-stage TNBC patients treated with neoadjuvant chemotherapy, complete disease regression was associated with high TIL expression, decreased PD-L1 expression, and low levels of FoxP3^+^ Treg cells [111].

During treatment of BC patients, the presence of a high level of Treg cell tumor infiltration has been linked to disease progression. Hence, Treg cell frequency could represent a predictive marker of therapy efficacy [112]. An increase in Treg cells observed after DC vaccine therapy can contribute to the inhibition of tumor regression [113]. In a murine BC model, this problem has been overcome by using a DC vaccine in association with an inhibitor of FoxP3 [114]. The positive effects of some anti-cancer therapies can also derive from a reduction in Treg cell levels in tumor tissues. Chloroquine, an anti-malaria drug, exerted anti-cancer activity in a murine BC model by reducing Treg cells and increasing CD8^+^ T cells [115]. The regression of a breast tumor model after treatment with artemisinin was associated with an increase of Th1 cells and a reduction of Treg cells [116].

Disease improvement observed after treatment of ER-α negative BC with aromatase inhibitors is associated with a decrease of Treg cells [117].

A high expression level of CD25 on Treg cells leads to an increase in their IL-2 consumption, causing IL-2 depletion that results in effector T cells’ reduced activity and apoptosis induction [118]. Treatment of HER2^+^ metastatic BC patients with IL-2 and trastuzumab did not induce tumor regression because of the absence of NK cell expansion [119].

In the complex, given the critical role played by Treg cells in producing an immunosuppressive TME, it is very important that focused studies aim to find novel therapeutic strategies to revert and reduce the effects of these immune cells.

### 1.2. Tumor-Associated Macrophages: M1 and M2 Phenotypes

Macrophages, which are pivotal players in the innate immune response, exert their influence by engaging in phagocytosis to eliminate cellular debris, regulating adaptive immunity, and contributing to inflammation resolution [120].

**Figure 2:**
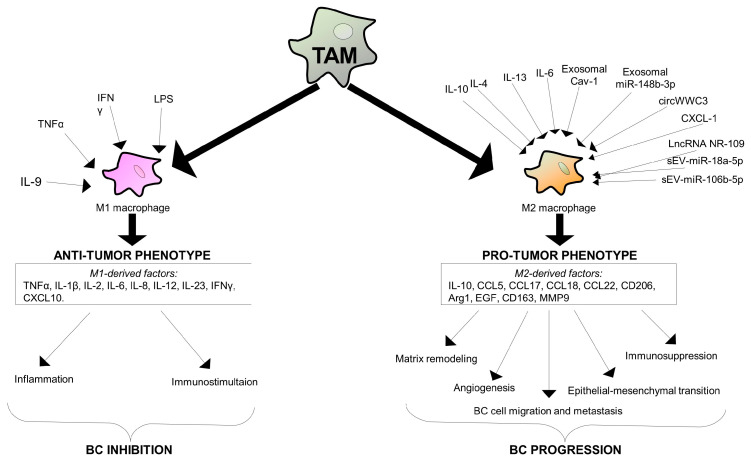
A schematic representation of tumor-associated macrophages (TAMs) in BCs. TAMs represent one of the main tumor-infiltrating immune cell types and can be grouped into two functionally different subtypes: M1 macrophages displaying an anti-tumor phenotype, and M2 macrophages displaying a pro-tumor phenotype. TAMs are forced to differentiate into M1 macrophages under the stimuli of specific proteins released in the tumor microenvironment, such as IL-9, TNFα, IFNγ and LPS. M1 macrophages produce massive amounts of cytokines and chemokines, represented by TNFα, IL-1β, IL-2, IL-6, IL-8, IL-12, IL-23, IFNγ, and CXCL10 and sustain inflammatory processes and immunostimulation. Conversely, M2 macrophage differentiation is induced by secreted factors such as IL-4, IL-10, IL-3, IL-6, circWWC3, and CXCL-1, molecule-derived vesicles repsuch as exosomal Cav-1, exosomal miR-148b-3p, sEV-miR-18a-5p, and sEV-miR-106b-5p, and long-noncoding RNAs such as LncRNA NR-109. The main M2 macrophage-derived factors are represented by IL-10, CCL5, CCL17, CCL18, CCL22, CD206, Arg1, EGF, CD163, and MMP9. M2 macrophages are involved in matrix remodeling, angiogenesis induction, epithelial–mesenchymal transition, immunosuppression, and BC cell migration and dissemination.

Within the intricate context of solid tumor tissues, TAMs emerge as immune cells infiltrating these environments. TAMs derived from monocytes and undergoing differentiation into macrophages during inflammatory processes [121] manifest in two main distinct phenotypes: M1 macrophages and M2 macrophages [122]. This duality underscores the complexity of their role within the TME, where they navigate between pro-inflammatory and anti-inflammatory functions. As TAMs play a dual role in both promoting and inhibiting tumor progression, understanding the delicate balance between M1 and M2 phenotypes holds significant implications for unraveling the broader dynamics of cancer inflammation and immunity that are associated with the BC metastatic process (Figure 2).

M1 macrophages exhibit pro-inflammatory and anti-tumor phenotypes associated with cytotoxicity and phagocytosis. These inflammatory cells play a pivotal role in the immune response by secreting a multitude of cytokines and chemokines, including TNFα, IL-1β, IL-2, IL-6, IL-8, IL-12, IL-23, IFNγ, and CXCL10. This diverse array of signaling molecules regulates and sustains inflammation, immunostimulation, and anti-cancer activity [122,123,124]. Conversely, M2 macrophages assume a distinct role in the immune landscape, synthesizing cytokines, chemokines, and proteins such as IL-10, CCL5, CCL17, CCL18, CCL22, CD206, Arg1, epidermal growth factor (EGF), CD163, and matrix metalloproteinase 9 (MMP-9). M2 macrophages are primarily involved in processes related to tissue repair, matrix remodeling, immunosuppression, and angiogenesis, contributing to a microenvironment that supports tumor progression [122,123,124]. In particular, the M2 phenotype is closely linked to several processes that drive BC progression, including increased epithelial–mesenchymal transition, extracellular matrix remodeling, angiogenesis, and dissemination [125,126], thus playing a crucial role in supporting tumor growth and metastasis. To this end, they produce anti-inflammatory factors and foster immune escape mechanisms sustaining cancer cell expansion [127,128].

One of the main mechanisms through which M2-macrophages exert their influence is by the induction of CD8^+^ T cell unresponsiveness. This process occurs through the dysregulation of the TCR pathway, and it emphasizes the complex interplay between immune cells and the TME in shaping the anti-tumor immune response [129].

TAMs exhibit the ability to differentiate into M1 or M2 subtypes in response to various external signals [130]. The differentiation of the M1 phenotype involves Th1 immune responses and is sustained by factors such as TNFα, IFNγ, and lipopolysaccharide (LPS) [131,132]. In vitro studies have revealed that IL-9-IL-9R signaling plays a role in the differentiation and proliferation of M1 macrophages. In fact, treatment with IL-9 triggers the transition from the M2 to the M1 phenotype, showcasing the dynamic nature of macrophage polarization [133]. Notably, M1 macrophages induced by IL-9 recruit anti-tumor immune cells into the tumor tissue through the chemoattractant macrophage chemokines CCL3/4 and CXCL9/10, thereby triggering anti-tumor immunity [133].

Conversely, M2 macrophage polarization is influenced by cytokines such as IL-4, IL-10, and IL-13, subsequently contributing to processes like tissue remodeling, immunosuppression, and angiogenesis [131]. In this context, KLF14, a member of the KLF family, emerges as a crucial regulator of the immune system and tumor development [134]. In particular, KLF14 has been identified as a key factor restraining tumor proliferation and invasion in colorectal cancer [135]. Notably, work by Jian Chu et al. has revealed that the KLF14 gene undergoes methylation in BCs, resulting in downregulation [136]. On the other hand, overexpression of KLF14 suppresses both BC proliferation and invasion capabilities, as observed in both in vitro and in vivo settings [136]. The specific mechanisms underlying these effects involve KLF14’s capacity to reduce the polarization of M2 macrophages. This is achieved by inducing SOCS3 transcription and inhibiting the RhoA/Rock/STAT3 signaling pathway [136].

MicroRNAs (miRNAs), circular RNAs (circRNAs), and long non-coding RNAs (lncRNAs) represent RNA molecules influencing M2 macrophage polarization. In particular, miRNAs play a crucial role in orchestrating inflammation and immunity [137], and among them miR-382 emerges as a notable regulator exerting control over the polarization of M2 macrophages and mitigating the metastatic capabilities of BCs. This regulatory effect is achieved by targeting peroxisome proliferator-activated receptor-gamma coactivator (PGC-1α) in TAMs and leading to a decrease in mitochondrial function [138]. Conversely, miR-148b-3p, found in abundance in MDA-MB-231 cell exosomes, drives the polarization of M2 macrophages by targeting tuberous sclerosis complex 2 (TSC2) and inhibiting the mTORC1 signaling pathway [139]. Functionally, circRNAs are either oncogenes or tumor suppressors, playing a crucial role in the intricate regulation of tumor growth [140,141]. Notably, circWWC3 has been implicated in driving BC progression by sustaining M2 polarization. CircWWC3 also contributes in vivo to the upregulation of PD-L1 expression in both TAMs and BC cells through the elevation of IL-4 expression [142].

LncRNAs are a class of RNA molecules that are longer than 200 nucleotides and do not encode proteins. LncRNAs participate in the regulation of gene expression at multiple levels and contribute to cell differentiation, immune responses, and cancer. Interestingly, the expression of lncRNA NR_109 is upregulated in M2-like macrophages within the TME of gastric and breast cancers, correlating with poor clinical outcomes of both tumor types and sustaining a pro-tumorigenic phenotype through the NR_109/FUBP1/c-Myc positive-feedback loop [143].

It is known that the stiffness of the ECM dramatically influences various aspects of cell behavior, including M2 macrophage polarization and that it contributes to the maintenance of the pro-tumor M2 phenotype. This effect is mediated through the induction of colony-stimulating factor 1 (CSF-1) production. CSF-1, in turn, plays a crucial role in macrophage recruitment, highlighting the intricate connection between mechanical cues from the ECM and the modulation of the TME [144]. Caveolin-1 (Cav-1), a protein associated with caveolae, plays a crucial role in regulating intracellular signaling and the metastatic process [145]. In BCs, Cav-1 within BC-derived exosomes has been found to induce the expression of genes associated with the premetastatic niche (PMN) in lung epithelial cells and to sustain M2 polarization of lung macrophages [146]. Moreover, Cav-1 represses the PTEN/CCL2/VEGF-signaling pathway, thereby contributing to the maintenance of M2 polarization [146].

### 1.3. Tumor-Associated Macrophages and Breast Cancer Metastasis

BCs exhibit the capacity for metastasis to various organs, including bones, lungs, brain, lymph nodes, and liver. Notably, lung metastasis is particularly associated with high mortality rates [147]. In the metastatic process primary tumors can induce the development of microenvironments in secondary tumor sites. These microenvironments play a crucial role in supporting the survival and outgrowth of cancer cells, forming pro-tumorigenic premetastatic niches that facilitate the extravasation and colonization of circulating cancer cells in secondary organs [148,149,150]. A key aspect in the constitution of metastatic niches is ECM remodeling, which has been identified as a crucial point in the metastatic process [151,152]. In particular, M2-TAMs that are CD206/CD163 positive are associated with lymph node metastasis and tumor size. These M2-TAMs play a role in inducing epithelial–mesenchymal transition by remodeling the extracellular matrix and promoting angiogenesis [125]. Notably, the secretion of fibroblast specific protein-1 (FSP-1) by macrophages supports the formation of BC metastasis by remodeling the ECM in the premetastatic niche [153]. TAMs are also a considerable source of EVs capable of sustaining cancer cell migration and invasion [154] and significantly regulating the communication between the BC primary site and the TME of the metastatic site [155].

In a murine experimental model, TAMs release exosomal miR-223-3p, which specifically supports the pulmonary metastasis of 4T1 cells [156]. In addition to TAM-derived EVs, recent work by Tang et al. demonstrated both in vitro and in a TNBC murine experimental model that BC small extracellular vesicles (sEVs) carrying miR-106b-5p and miR-18a-5p trigger M2 polarization and PD-L1 expression in macrophages [157]. This regulatory effect involves the modulation of PTEN/AKT/PD-L1 and PI-AS3/STAT3/PD-L1 pathways, leading to an increased capability of BC cell invasion and metastasis [157].

TNBC tissues have significant infiltration of polarized TAMs, particularly of the M2-like subtype. Intriguingly, M2-like macrophages in TNBC tissues sustain EMT and foster the development of cancer stem cell (CSC) [158] through the CCL2/AKT/β-catenin axis, which can represent a potential TNBC therapeutic target [158].

EMT stands out as a pivotal process in the metastatic cascade, and the methyltransferase DNMT1 emerges as a key regulator of gene expression as it is upregulated in various malignancies [159,160]. Zhongwei Li et al. have identified [161] the IL-6-pSTAT3-ZEB1-DNMT1 axis as a crucial regulator of TAM-induced BC metastasis. The transcription factor ZEB1, a component of this axis, plays a dual role by inhibiting the expression of E-cadherin and inducing EMT [162,163]. ZEB1, in turn, sustains the expression of DNMT1, and TAMs, during their interaction with BC cells, enhancing DNMT1 expression through the IL-6-pSTAT3-ZEB1-DNMT1 axis. Upregulation of DNMT1 is essential for cancer cell migration and in vivo metastasis [161]. Importantly, EMT has also been implicated in inducing alternative splicing, adding another layer of complexity to the metastatic process [164,165]. These findings shed light on the intricate molecular mechanisms orchestrated by the IL-6-pSTAT3-ZEB1-DNMT1 axis in TAM-induced BC metastasis, emphasizing potential targets for therapeutic interventions aimed at disrupting key steps in the metastatic cascade.

BCs display several epigenetic alterations, such as DNA methylation regulating gene expression and contributing to oncogenesis [166]. Interestingly, decitabine (DAC), an FDA-approved inhibitor of DNA methyltransferase that is primarily used for hematologic diseases, inhibits BC cell migration that is sustained by TAMs [167,168]. The promising inhibitory effect of DAC on BC cell migration mediated by TAMs suggests its potential as a therapeutic agent for targeting metastatic processes [161].

The immunological landscape undergoes a notable shift from BC primary sites to metastatic sites, which exhibit reduced immunological activity [169]. A key immunological distinction between primary tumor and metastatic sites is the increased presence of M2 macrophages in metastases [169]. Notably, the acetyl-lysine reader cat eye syndrome chromosome region candidate 2 (CECR2), known for its role in chromatin remodeling, is upregulated in metastases with respect to primary tumors. CERC2 upregulation is associated with M2 macrophage increases and correlates with worse metastasis-free survival outcomes [169,170]. Interestingly, treatment with a CECR2 inhibitor restrains M2 polarization induced by cancer cells and suppresses cancer cell metastatic potential [169].

It has been found that macrophages express RON receptor tyrosine kinase (MST1R) that is upregulated or constitutively activated in >50% of human BCs [171] Moreover, MST1R expression in BC tissues is linked to cancer metastasis and poor prognosis irrespective of the molecular subtype [172]. Interestingly, the loss of RON expression in macrophages inhibits mammary tumor cell self-renewal, proliferation, survival, and migration, and leads to reductions in tumor growth and metastatic outgrowth [173]. These findings highlight the intricate relationship between MST1R expression, macrophage function, and BC progression, emphasizing its potential as a target for therapeutic intervention to modulate the TME and restrain metastatic processes.

### 1.4. Myeloid-Derived Suppressor Cells

**Figure 3:**
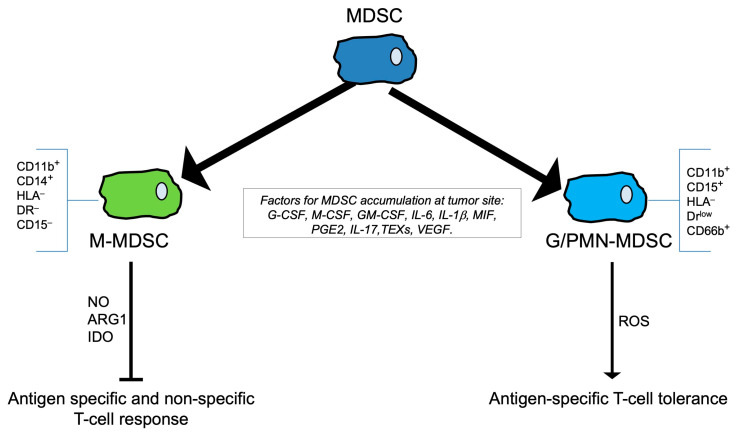
Schematic representation of myeloid-derived suppressor cells (MDSCs). MDSCs are a heterogeneous population of immune cells that play a crucial role in regulating immune responses and maintaining immune homeostasis. Two main categories of MDSCs exist: monocytic MDSCs (M-MDSCs) that are endowed with the typical morphology of monocytes, and granulocytic or polymorphonuclear MDSCs (G-MDSCs or PMN-MDSCs, respectively), which are more similar to granulocytes. M-MDSCs are CD11b^+^CD14^+^HLA^−^DR^−^CD15^−^ whereas G-MDSCs are CD11b^+^CD15^+^HLA^−^DR^low^CD66b^+^. The accumulation of MDSCs at a tumor site is regulated by some pivotal factors (G-CSF, M-CSF, GM-CSF, IL-6, IL-1b, MIF) produced by tumor cells or stromal cells in response to inflammation and chronic infection. MDSCs contribute to cancer progression by altering the microenvironment to support tumor growth and by facilitating immune evasion. In particular, M-MDSCs release elevated levels of suppressive factors, including NO, ARG1, and IDO, which enable them to inhibit both antigen-specific and -non-specific T cell responses. On the other hand, PMN-MDSCs generate high levels of ROS, inducing antigen-specific T cell tolerance.

Myeloid-derived suppressor cells (MDSCs) represent a heterogeneous population of immune cells that play a crucial role in regulating immune responses and maintaining immune homeostasis. Moreover, MDSCs have gained significant attention in recent years due to their immunosuppressive properties and their involvement in various pathological conditions. MDSCs arise from the myeloid lineage and are defined on the basis of their morphology, surface markers, and functions. Two main categories of MDSCs exist: monocytic MDSCs (M-MDSCs), which are endowed with the typical morphology of monocytes, and granulocytic or polymorphonuclear MDSCs (G-MDSCs or PMN-MDSCs, respectively), which are more similar to granulocytes [174,175]. M-MDSCs are CD11b^+^CD14^+^HLA^−^DR^−^CD15^−^ whereas G-MDSCs are CD11b^+^CD15^+^HLA^−^DR^low^CD66b^+^. In addition, a novel group of MDSCs has recently been defined, namely early-stage-MDSCs (e-MDSCs), which represent an immature group of MDSCs with Lin^−^HLA^−^DR^−^CD33^+^ as markers [175] (Figure 3).

The development, the expansion, and the status of activation as well as the migration of MDSCs is regulated by a wide variety of cytokines and chemokines. It is possible to distinguish the factors that are responsible of the accumulation and recruitment of MDSCs at the tumor site from those that induce the status of activation of MDSCs.

A wide variety of signaling pathways and regulators, including STAT family members, interferon cascade regulators, adenosine receptor A2b, and inflammasome members such as NLRP3 collectively play pivotal roles in stimulating myelopoiesis, suppressing the maturation and differentiation of progenitor cells, and facilitating the expansion of immature myeloid cells [176,177]. Further, the factors responsible for triggering the pathological activation of immature cells that adopt an immunosuppressive phenotype encompass a range of signaling pathways and regulators, including the NF-κB pathway, STAT1 pathway, STAT6 pathway, prostaglandin E2 (PGE2), COX-2, and the ER stress response pathway [176,177].

Granulocyte colony-stimulating factor (G-CSF) [178,179], macrophage colony-stimulating factor (M-CSF) [178,179], GM-CSF [178,179], IL-6 [180], IL-1β [181], macrophage migration inhibitory factor (MIF) [182], polyunsaturated fatty acids, PGE2 [183,184], IL-17 [185], miRNAs derived from tumor exosomes (TEXs) [186,187], and vascular endothelial growth factor (VEGF) [188], which are produced by tumor cells or stromal cells in response to inflammation and chronic infection, are pivotal factors for the accumulation of MDSCs at tumor site. Moreover, for the recruitment of MDSCs to specific inflammatory sites, chemokines such as C-X-C motif chemokine ligand (CXCL)5, C-C motif chemokine ligand (CCL)1, CCL2, CCL5, and MCP1, along with the proteins S100 calcium-binding protein A8 and A9, are essential [189].

BCs exert regulatory control over the production of chemokines by MDSCs through various pathways. Elevated secretion of CXCL1 by lung fibroblasts diminishes the immune response within the lung microenvironment, leading to the recruitment of G-MDSCs and promoting the development of microenvironmental niches conducive to anterior lung metastases in BCs [190].

Upon the activation of specific transcriptional factors, MDSCs can differentiate into TAMs and inflammatory dendritic cells (inf-DCs), whereas G-MDSCs, which have a shorter half-life, differentiate into tumor-associated neutrophils (TAN) [191]. The development and progression of BCs involve a transition from MDSCs to TAMs. Within MDSCs, IL-33 promotes the expression of IL-13 while inhibiting that of IL-12. Consequently, this polarization sustains the development of M2 macrophages and Th2 cells within the TME, posing a detriment to anti-tumor immunity [192].

MDSCs play a significant role in the suppression of tumor-fighting T and B cells, especially CTLs, as well as of pro-inflammatory cells such as NK cells within the TME [193]. Furthermore, MDSCs contribute to cancer progression by inducing the formation of Treg cells and Th17 cells, thereby altering the microenvironment to support tumor growth and facilitating immune evasion [191]. Beyond their immunosuppressive functions, MDSCs also affect BC progression through non-immunosuppressive pathways, including the promotion of tumor stem cells, mediation of EMT, and stimulation of angiogenesis. The accumulation of MDSCs driven by TME results in immunosuppression through intricate mechanisms [194,195,196], with different MDSC subsets employing distinct mechanisms to induce immunosuppression in various tissues. In peripheral lymphoid organs, PMN-MDSCs generate high levels of reactive oxygen species (ROS), inducing antigen-specific T cell tolerance. In contrast, M-MDSCs release elevated levels of other suppressive factors, including nitric oxide (NO), ARG1, and IDO, which enable them to inhibit both antigen-specific and non-specific T cell responses. Furthermore, M-MDSCs maintain heightened levels of activated STAT3, which obstructs their differentiation into dendritic cells (DCs) or macrophages. However, at tumor sites, hypoxic stress dramatically reduces activated STAT3 levels in M-MDSCs, leading to their differentiation into TAMs. ROS production in PMN-MDSCs at tumor sites decreases, whereas ARG1 and other suppressive factors, such as IDO and prostaglandin E2 (PGE2), which are produced by PMN-MDSCs, drive non-specific T cell suppression. It is worth noting that PMN-MDSCs at tumor sites have a reduced lifespan, and that molecules released by dying PMN-MDSCs can still induce immunosuppression [194,197,198]. Another mechanism of immunosuppression is represented by the effect of MDSCs on lymphocyte migration. In particular, in peripheral lymphoid organs MDSCs dampen the immunological response and accumulate in sentinel lymph nodes (LNs), where they impede CD3/CD28-induced T cell proliferation through contact-dependent mechanisms. This, in turn, promotes tumor development and metastasis [199,200]. Hanson E.M. et al. have revealed that MDSCs in BC downregulate L-selectin expression on the surface of CD4^+^ and CD8^+^ T cells by producing ADAM17 (a disintegrin and metalloproteinase domain 17) on the plasma membrane. This action restricts the activation and entry of naive T cells into LNs and their trafficking to tumors, ultimately suppressing the anti-tumor immune response [201]. Further, there is a significant upregulation in PD-1 expression in MDSCs, and several studies have linked various mediators in the TME, such as LPS, to the induction of PD-1 expression in MDSCs within BCs [202]. Through the PD-1 and PD-L1 axis, MDSCs have been shown to enhance immune evasion mediated by PD-1/PD-L1 B-reg cells by activating the phosphatidylinositol 3-kinase (PI3K)/protein kinase B (AKT)/NF-κB signaling pathway in B cells [203]. Within BCs, MDSCs play a pivotal role in promoting tumor progression and metastasis through a multifaceted mechanism. They not only exert immunosuppressive effects, thereby weakening the body’s natural anti-tumor immune response and facilitating BC growth and metastasis, but they also undermine the effectiveness of various therapeutic interventions. Currently, a range of MDSC-targeted therapies, including immunotherapy and combination treatments with conventional therapies such as chemotherapy and radiation therapy (RT), are undergoing preclinical evaluation to assess their potential in enhancing anti-tumor effects in BCs. We anticipate that a deeper understanding of the clinical significance of MDSCs will stimulate further research and development of MDSCs-targeted therapies, ultimately improving prognoses for BC patients.

### 1.5. Conclusions

**Figure 4:**
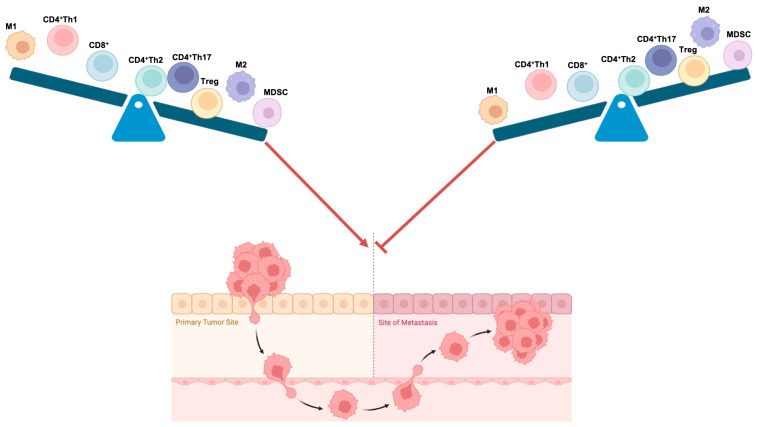
A schematic representation of the influences of TILs, TAMs, and MDSCs on breast cancer metastasis. The figure depicts the immune cell population balance that affects the metastatic capability of breast cancer cells. Arrow indicates induction and T-bar represents inhibition. Created with BioRender.com.

TME plays a pivotal role in influencing metastatic processes and therapeutic outcomes in all BC subtypes. Comprising non-cancerous stromal cells, immune cells (such as tumor-infiltrating lymphocytes, macrophages, and myeloid-derived suppressor cells), ECM, and blood/lymphatic vessels, the TME dynamically regulates the progression of BCs. In particular, CD8^+^ cytotoxic T cells, CD4^+^ T cells, TAMs, and MDSCs emerge as major players in shaping the immunological milieu within the BC microenvironment. Furthermore, the interaction between TME components and the cross talk of TME with cancer cells generate a tumor niche that triggers immunosuppressive processes leading to BC dissemination. Understanding these intricate interactions in BCs holds significant promise for the development of targeted therapeutic strategies in medical oncology. Therefore, the aim of this article has been to describe the roles of TILs, TAMs, and MDSCs in breast cancers, as well as the factors and mechanisms by which every immune cell population regulates breast cancer progression. In particular, this review has attempted to explore the pivotal roles of T lymphocytes, macrophages, and MDSCs in maintaining homeostasis within the BC immune microenvironment and influencing the metastatic cascade (Figure 4). In the complex, this article reviews recent insights concerning the breast immune microenvironment that can contribute to the development of new therapeutic approaches aimed at sustaining anti-tumor immunity and restraining metastatic progression in BCs. However, we believe that immune TME phenotype studies aiming to revert the immune-suppressive TME of BCs, as well as the characterization of signaling affecting the plasticity of immune TME, could be useful in developing therapeutic advances in BC research.
